# Ecological risk assessment on heavy metals in soils: Use of soil diffuse reflectance mid-infrared Fourier-transform spectroscopy

**DOI:** 10.1038/srep40709

**Published:** 2017-02-13

**Authors:** Cheng Wang, Wei Li, Mingxing Guo, Junfeng Ji

**Affiliations:** 1 Jiangsu Key Laboratory of Atmospheric Environment Monitoring and Pollution Control, Collaborative Innovation Center of Atmospheric Environment and Equipment Technology, School of Environmental Science and Engineering, Nanjing University of Information Science & Technology, Nanjing, 210044, China; 2 Key Laboratory of Surficial Geochemistry, Ministry of Education, School of Earth Sciences and Engineering, Nanjing University, Nanjing 210093, China; 3 Department of Agriculture & Natural Resources, Delaware State University, Dover, DE 19901, USA

## Abstract

The bioavailability of heavy metals in soil is controlled by their concentrations and soil properties. Diffuse reflectance mid-infrared Fourier-transform spectroscopy (DRIFTS) is capable of detecting specific organic and inorganic bonds in metal complexes and minerals and therefore, has been employed to predict soil composition and heavy metal contents. The present study explored the potential of DRIFTS for estimating soil heavy metal bioavailability. Soil and corresponding wheat grain samples from the Yangtze River Delta region were analyzed by DRIFTS and chemical methods. Statistical regression analyses were conducted to correlate the soil spectral information to the concentrations of Cd, Cr, Cu, Zn, Pb, Ni, Hg and Fe in wheat grains. The principal components in the spectra influencing soil heavy metal bioavailability were identified and used in prediction model construction. The established soil DRIFTS-based prediction models were applied to estimate the heavy metal concentrations in wheat grains in the mid-Yangtze River Delta area. The predicted heavy metal concentrations of wheat grain were highly consistent with the measured levels by chemical analysis, showing a significant correlation (r^2^ > 0.72) with acceptable root mean square error RMSE. In conclusion, DRIFTS is a promising technique for assessing the bioavailability of soil heavy metals and related ecological risk.

Soil contamination by heavy metal has been common worldwide owing to the rapid industrialization and urbanization. Depending on the level of pollution, the adverse effects of soil heavy metals on plant growth, crop productivity, and food safety can be disastrous. A substantive challenge for assessing the ecological risk of heavy metal-contaminated soils is to set a simple, rapid and practical technique for predicting the concentrations of heavy metals accumulating in the growing plants, especially in the edible tissues^1^. It is well known that the biotoxicity and phytoaccumulation of heavy metals in soil, though varying with plant species, are not determined by their total concentrations but by their bioavailability, which is influenced by the metal species, metal affinity for plant roots, existing forms of metals in soil, and the soil properties including pH, organic matter composition, and the presence of other cations and anions^2^,^3^,^4^. To predict the bioavailability of heavy metals in soil for a specific plant, soil properties and other influencing environmental factors have to be taken into account. Various chemical analytical methods have been developed to estimate the “bioavailable concentration” of heavy metals in soil, including the chemical extraction, and pore water analysis^5^. The analytical results of chemical extraction are necessarily validated by correlating to plant test outcomes heavy metal bioaccumulation and biotoxic symptoms via linear regression analysis^5^. Nevertheless, chemical analysis is time consuming and can be costly. Furthermore, the established interrelationship is typically soil-specific: the correlation shifts among different soils, as soil properties strongly influence the bioavailability of heavy metals^6^,^7^. In addition, the established correlation varies with the floristics and development stage of plants^1^.

Infrared (IR) spectroscopy is a powerful analytical technique for qualitatively identifying and quantitatively measuring characteristic functional structures of various chemicals. The method is rapid, non-destructive, and non-hazardous, requiring minimal sample treatment (e.g., mostly simple grinding)^8^,^9^. In particular, the near infrared (NIR, with the wavelength ranging from 400 to 2500 nm or the wave number from 25000 to 4000 cm^−1^) spectra of a sample illustrate the overtones and combinations of vibrational bands of light atoms that have strong molecular bonds, whereas the mid infrared (MIR, with wavelength of 2500–25000 nm or wave number of 4000–400 cm^−1^) spectra reflect the fundamental vibration patterns of the chemical bonds C–H, N–H, O–H, C–O, Si–O and so on^10^,^11^ and therefore, can provide comprehensive information about the chemical composition of the test sample^9^,^12^. The NIR spectroscopy was explored for estimating the heavy metals concentrations of soils by a number of researchers^8^,^14^. The results, however, were not promising, as NIR failed to sufficiently detect heavy metals in soil, and causing many scientists to question the feasibility and perspective of the method^15^,^16^. In contrast, the MIR reflectance spectra are able to reveal essential information related to both organic bonds and inorganic components in soil samples^8^,^17^, and illuminate distinct spectral features of the inherent heavy metals. For MIR spectrometers using the Fourier-transform technology, this analytical technique is known as Diffuse Reflectance mid-Infrared Fourier-Transform Spectroscopy (DRIFTS). The DRIFTS has been investigated over the past fifteen years for qualitative and quantitative analyses of soil matrices for organic, nitrogen, and heavy metal components. The technique involves regression analysis of soil spectral data from MIR scans and against concentration data from chemical analysis to identify the spectral information (i.e., peaks) closely related to the targeted soil parameters (e.g., nitrogen content) and further calibration analysis of the targeted soil parameter at concentration gradients against the extracted spectral information to establish a predictive equation. The predictive equation can then be followed to estimate the effective value of the targeted soil parameter of any regional samples based on their MIR spectra; no chemical analysis is needed^13^,^18^. By using DRIFTS, research has shown that specific markers existed in soil MIR spectra for organometallic complexes and minerals^19^. Furthermore, the influences of soil physical and chemical properties on the metal activity were also incorporated in the spectra^18^,^20^,^21^. Successful applications of DRIFTS to estimate soil nitrate, sulfate, organic carbon, and organic nitrogen contents have been reported^22^,^23^,^24^,^25^,^26^. Recent studies indicate that the technique can be applied to detect heavy metals and their chelation with anionic functional groups in soil^10^,^11^,^13^. By testing 4,130 European agricultural soils, Soriano-Disla *et al*.^10^,^11^. concluded that DRIFTS was an effective method capable of predicting the concentrations of heavy metals in soil.

Considering that the bioavailability of heavy metals in soil is largely governed by the interactions (chelation, sorption etc.) between the metal species and existing anionic functional groups, the DRFITS technique may be a potential option to effectively and integratively estimate soil heavy metal bioavailability. This study aimed to explore an approach of predicting bioaccumulation of soil heavy metals (Cd, Pb, Hg, As, Ni, Cr, Cu, Zn and Fe) in wheat grain based on the soil DRIFTS spectral information.

The Yangtze River Delta in China was chosen as the study area. The area is an alluvial flood plain with concentrated industry and intensified economy. Cultivation agriculture is intensive in the region, with winter wheat as a staple crop in addition to rice. The study area is characterized by a typical subtropical monsoon climate with the average annual precipitation of 1,000–1,400 mm and the average annual evapotranspiration of 950–1100 mm. The soil in the region was developed from marine strata and fluvial alluvial deposits, and has been suffering anthropogenic acidification^27^. The rapid industrial development in the recent thirty years has transformed the area from a traditional agricultural arena to a prosperous urban, industrial and agricultural agglomeration. This rapid development has also generated substantial environmental problems, for example, the introduction of external heavy metals to cropland soil and the incidences of severe soil pollution by heavy metals at many localities^28^,^29^. In addition, the soil in the region covers a rather wide range in the major quality parameters^30^, making the area an ideal experimental field to investigate the relationship between soil MIR spectra and the bioavailability of soil heavy metals (as indicated by concentrations of heavy metals in the cultivated wheat).

We first established the DRIFTS-based calibration models for predicting heavy metal concentrations of wheat grain using soil DRIFTS spectra and chemically-measured wheat grain metal concentration data of 126 paired soil and grown wheat grain samples from the Yangtze River Delta region. In consideration of the food safety standards of China, the established prediction models were applied to assess the ecological risks of Cd in the regional soil as represented by a new batch of 154-sample collection. Finally, we chemically analyzed the concentrations of Cd in 24 wheat grain samples collected in the later year from grown the pre-assessed high risk zones, and validated the established metal bioavailability prediction models and the corresponding risk assessment by comparing the model-predicted and the chemically-determined wheat grain Cd concentration results. The research contributes significantly to establishing a rapid, practical, cost-effective and environmentally-friendly method for assessing the bioavailability of heavy metals in soils and the related food safety risk of agro-products grown in the soil. This research also confirms the feasibility of using the DRIFTS technique to early alarm the potential ecological risks of heavy metals in soils.

## Results and Discussion

### Soil properties

The soils in the study area were strongly acidic to moderately alkaline, with pH ranging from 4.8 to 8.3 (average 6.7). The concentrations of heavy metals, organic carbon (OC), N, P, K, S, Fe, Ca, Mg and Mn in the soils are presented in Table S-1 (SI) in the Supplementary Information. In general, the concentrations of heavy metals (except Cd) and other quality parameters of the tested Yangtze River Delta soils lay in the normal ranges of worldwide agricultural soils^31^. The average Cd concentration, however, was higher than the reported average for Chinese soils^30^. There were three soil samples containing Cd at concentrations exceeding the Chinese regulatory level (0.6 μg g^−1^) for cropland soils^32^.

### Soil DRIFTS spectra

The DRIFTS spectra of one soil (X-1) representative of the tested 126 samples are shown in Fig. S-1(SI). All the soil samples yielded reflectance spectra similar in shape yet different in peak intensity, in consistence with the findings by Siebielec *et al*.^13^. In the present study, the measured soil spectra are characterized by distinct absorption bands at near 698 cm^−1^, 814 cm^−1^, 924–1160 cm^−1^, 1614–2430 cm^−1^, and 3850–3250 cm^−1^, which are generally associated with molecular vibrations of the functional groups SO_3_^2−^, NO_3_^−^, Si−O, C = C (or –COO), and O−H, respectively. These absorption peaks indicate the presence of anionic and molecular functional groups of minerals in the test soils. The band located at 698 cm^−1^ is related to the presence of SO_3_^2−^, and at 814 cm^−1^ represent the presence of NO_3_^− ^22,23. The bands at 924–1160 cm^−1^ might be caused by the C−C of carbohydrates which with overlapping the bands corresponding to soil phyllosilicate^9^,^25^. The bands at 1640–1850 cm^−1^ could be caused by the C = C or –COO asymmetric stretching of metal carboxylates^33^,^34^,^35^. The peak at 2522 cm^−1^ is likely due to the complexation of carbonate with metal cations^12^.

### Heavy metal concentrations of wheat grain

Chemical analysis revealed that the wheat grains from the Yangtze River delta region contained 0.018–0.245 μg g^−1^ of Cd, 0.022−0.109 μg g^−1^ of Pb, 0.4−6.6 μg kg^−1^ of Hg, 0.070–1.106 μg g^−1^ of Ni, 0.04–0.37 μg g^−1^ of Cr, 4.02–17.75 μg g^−1^ of Cu, 20.9–108.6 μg g^−1^ of Zn, and 34.0–193.0 μg g^−1^ of Fe (the means are presented in Table S-1, SI). According to the Chinese Maximum Permissible Concentrations (MPC) for heavy metals in wheat grain^36^, the measured concentrations of Hg, Pb, Cr, Cu and Zn were under the regulatory levels, while 14.3% of the 126 wheat grain samples contained Cd exceeding the MPC level and 5.6% of the samples contained Ni at above the average Ni concentration of the world agricultural foods (0.8 μg g^−1^)^37^.

### Predicting heavy metal concentrations of wheat grain from soil DRIFTS spectra

**Figure 1:**
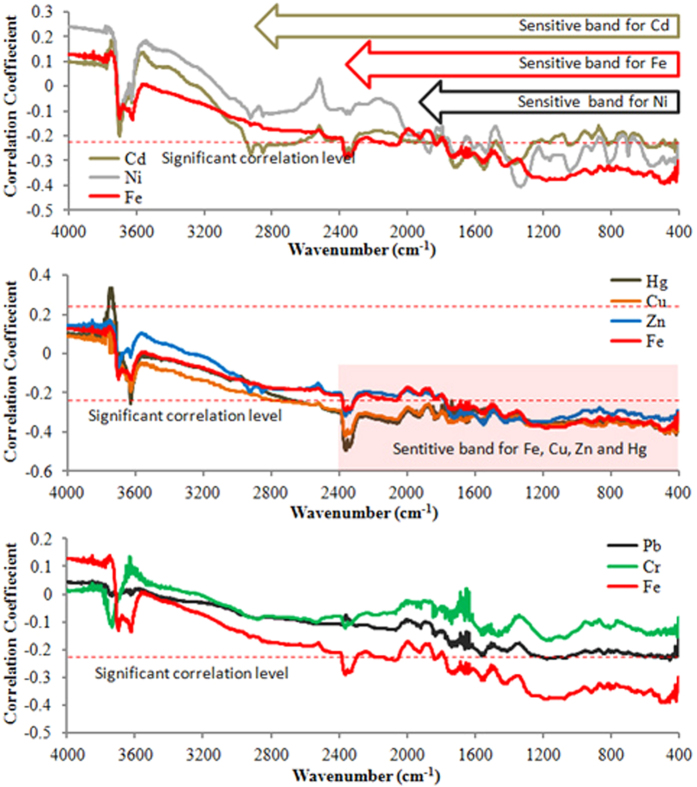
Coefficients of correlation between heavy metal concentrations of wheat grain and reflectance of soil DRIFTS. Reflectances at the 400–2400 cm^−1^ band were significantly correlated to the concentrations of Hg, Cu, Zn, Fe, Cd and Ni, respectively, but not significantly correlated to those of Pb and Cr. “Sensitive band for metal” refers to the band at which soil MIR reflectance shows significant correlation to the metal concentration of wheat grain. The sensitive band ranges are marked with hollow arrows in the first diagram (for Cd, Fe and Ni) and a pink rectangle in the second diagram (for Fe, Cu, Zn and Hg).

The correlation coefficients between the concentrations of heavy metals in wheat grain and the reflectance of soil DRIFTS are presented in Fig. 1. The reflectances at the band of 400–2400 cm^−1^ were significantly correlated to the concentrations of Hg, Cu, Zn, Fe, Cd and Ni in wheat grain, respectively. No significant correlations were observed for Pb and Cr. As a result, the sensitive bands of soil spectra for Fe, Cu and Zn in wheat grain were defined at 400–2400 cm^−1^, whereas at 400–2400 and 3530–3780 cm^−1^ for Hg, at 400–2980 cm^−1^ for Cd, and at 400–1920 cm^−1^ for Ni. The bands in the 400–1300 cm^−1^ region reflected the Si−O stretching and bending bands as well as and O−H bending bands from soil clay minerals. The bands at 400–600 cm^−1^ were also associated with the metal-carbon stretching of organometallic compounds^19^. The 650–1500 cm^−1^ bands were generally regarded as fingerprints of molecules. The bands at 1700–2130 cm^−1^ were due to metal−CO or metal−CO−metal^19^. The bands at 3530–3780 cm^−1^ may be associated to O−H and Si–O groups of silicates^25^.

**Figure 2:**
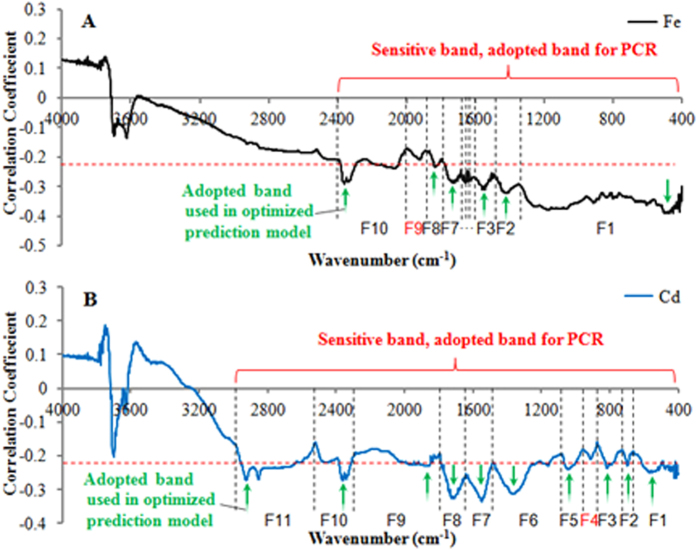
Results of principal component analysis (PCA) and the optimization of adopted spectral bands for prediction models. A total of 10 and 11principal components were extracted for Fe and Cd, respectively, based on the sensitive bands using PCA analysis. The corresponding band of each PC factor is indicated by a trough (peak value) of correlation coefficient. To optimize the prediction models, for each PC-corresponding spectral region, one representative band showing the highest correlation with metal concentration was chosen to establish the regression model. Simultaneously, reflectance of the chosen band should also significantly correlates to the heavy metal concentration of wheat grain (|R^2^| > 0.229, *p* < 0.01). Since the processes for Cu, Zn, Ni and Hg are consistent, the information about these metals is not reported.

Principal component analysis (PCA) of the soil spectra and wheat grain metal concentration data resulted in a 10-PC (principal component) for Fe, Cu and Zn, 11-PC for Cd and Hg, and 8-PC for Ni, with covering >99.0% of the variance data (Fig. 2 and Table S-2, SI). In all the case, the first three PCs account for more than 90.0% of the total variation. As such, the driving forces could be generalized to the three predominated factors. Each component refers to the soil reflectance at a specific spectral band. The bioavailability of heavy metal in soil depends on their complexation with anion functional groups. For example, metals in complexation with carbonate generally exhibit a rather low bioavailability. Soil MIR spectra are able to illustrate these complexations, but the information is overlapped with the effects from multiple factors, becoming rather sophisticated to distinguish. Based on this phenomenon, the most key information about the targeted variable (referring herein to the association between soil MIR spectra and the concentrations of heavy metal in wheat grain) was extracted by applying PCA to reduce dimensionality of a set of variables. These extracted PCs may be indicators of relevant soil parameters influencing heavy metal accumulation in wheat, such as silicate, organic matter, sulfate, nitrate, phosphate. Varying with the breadth of the adopted spectral bands, the elements Hg and Cd both required 11 PC in their prediction models, whereas the prediction model of Ni used fewer PCs (Fig. 2 and Table S-2, SI). The variations reflected the similarity and difference in geochemical behaviors of various heavy metals. In the soil-plant system, Cu and Zn demonstrate geochemical behavior rather similar to that of Fe, while Cd is more similar to Ca in geochemical processes^31^.

As shown in Table S-2(SI), the coefficients of determination (R^2^ and RMSE) for all predictions in linear regression analysis reached the significant level. However, if apply such these models in a field region, substantial work on data processing and statistical manipulation (e.g., PCA and linear regression to identify the correlationship between bioavailability of heavy metal and reflectance of the whole MIR band) must be carried out in advance. To facilitate the application of prediction models, the optimal and representative bands of PCs were first chosen in the present study, and then the wheat grain heavy metal concentration data were regressed against the reflectance of the representative bands of the PCs. The models for predicting concentrations of individual metal elements in wheat grain based on the reflectance of representative bands of soil DRIFTS are listed in Table 1. The R^2^ of all the model predictions were significant (*p* < 0.05), and better than those of the PCA predictions in Table S-2 (SI).

### Risk assessment on heavy metal bioavailability using soil DRIFTS-based prediction model

**Figure 3:**
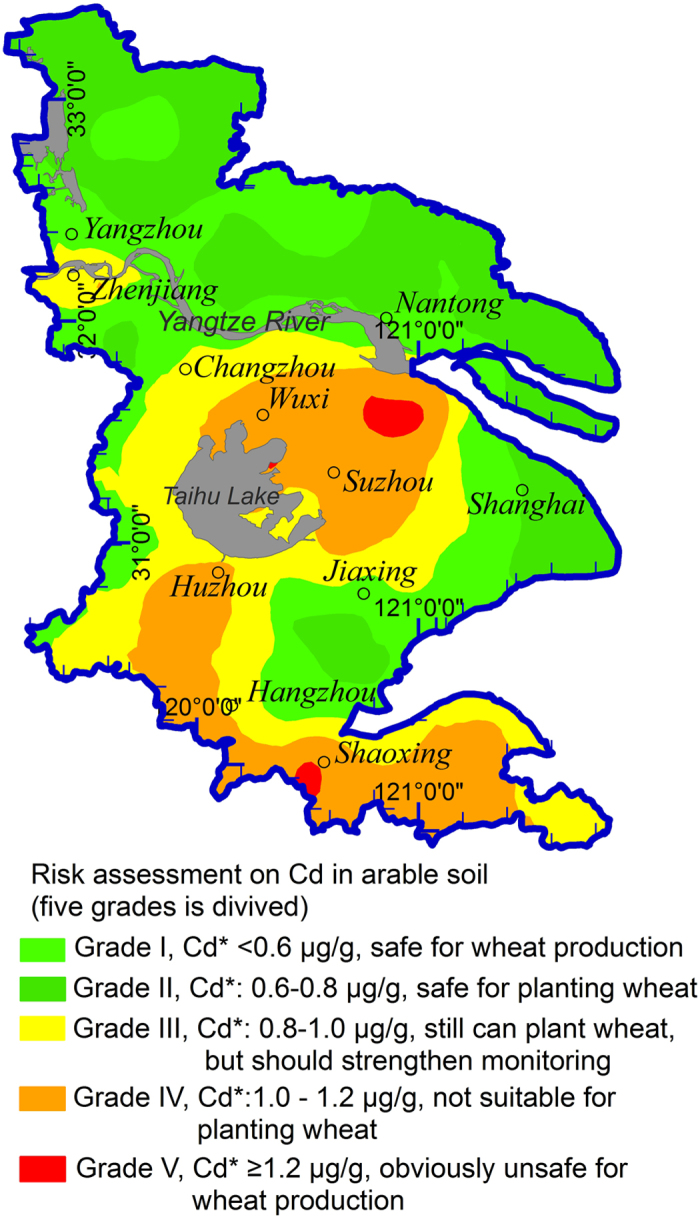
Spatial distribution of ecological risks assessment of Cd in arable soils. Cd* denotes the predicted Cd concentration of wheat grain. The grade division was based on the Chinese Maximum Permissible Concentration of Cd, for which five grades are classified. The “Grade IV” (color denoted by orange) means that the corresponding sites should be early-warned as “Not suitable for planting wheat”, and “Grade V” (color denoted by red) should be early-warned as “obviously unsafe for wheat production”. The map was created by software ArcGIS 9.3.

The established models in the Table 1 can be used to predict the concentrations of heavy metals in wheat by scanning the soil with a MIR device. In reference with the food safety standards for heavy metals in wheat grain, the most outstanding and significant application of the models is to assess the risk (and issue early warning) of the bioavailability of heavy metals in soil. The present study developed and tested such an approach by introducing the Chinese food safety standards for heavy metals in wheat grain^36^ and the models in Table 1 to the prediction and assessment of heavy metal concentrations in wheat grain. Taking Cd as an example, the soil DRIFTS spectral information was applies in the wheat grain heavy metal concentration prediction model (Table 1) to assess the ecological risk of Cd in soils (154 soil samples) of the Yangtze River Delta region. The results are presented in Fig. 3. The established approach divided the soils into five grades corresponding to the categorized concentration levels of Cd in wheat grain. The detailed process was narrated in Supporting Information. According to the assessment results, the soils in the Suzhou-Wuxi region (Middle Yangtze River Delta area) were not safe in terms of Cd level for wheat production. The Suzhou-Wuxi local area was then grouped into “Grade V”, meaning “obviously unsafe for wheat production”. In fact, the Suzhou-Wuxi region is a concentrated manufacture industry zone. The natural environment has been suffering from severe heavy metal pollution. Previous investigations had recognized that Cd pollution in the region was related to industrial emissions^28^,^30^.

### Model assessment

**Figure 4:**
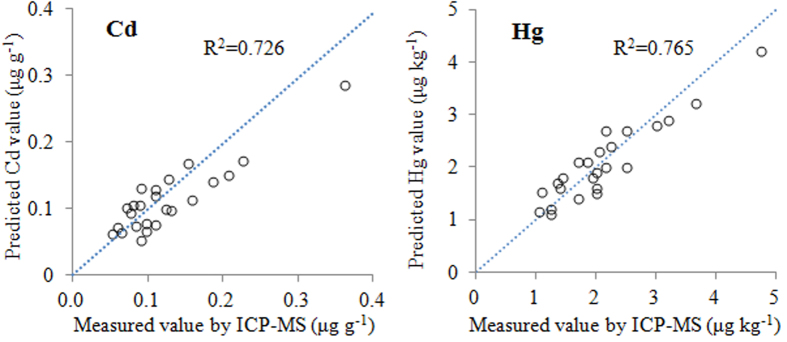


The prediction models were further assessed by comparing the prediction results with chemical measurements of Cd concentrations of wheat grain (24 samples) collected from the Middle Yangtze River Delta region. As shown in Fig. 4, a clear relationship existed between the two sets of paired data (R^2^ > 0.72), indicating that the models were effective for predicting bioaccumulation of heavy metals in wheat grain based on the soil DRIFTS spectral information.

**Figure 5:**
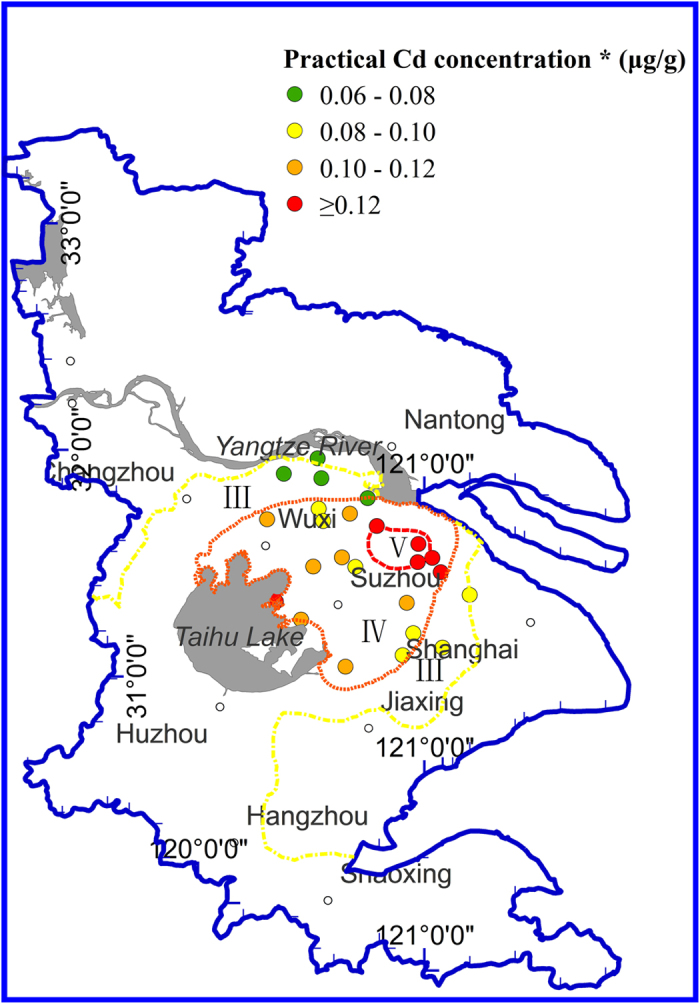
Comparison the chemically-measured concentrations of Cd in wheat grain versus the result of ecological risk assessment of Cd in soils (i.e., spatial distribution map of model-predicted Cd concentrations in wheat grain from the Yangtze River Delta region). Cd concentration* represents the chemically-measured Cd concentrations, and is denoted as filled circles; different filled colors denote varying risk grades (for example, red indicates that the Cd concentration of wheat grain reached or exceeded the threshold value of Grade V). According to the results of ecological risk assessment on Cd (Fig. 4 and Table S-3, SI), the wheat grown in arable soils in the Suzhou-Wuxi region will generate Cd in the wheat grain at three concentration grades (III, IV and V), with the corresponding zones delineated with yellow, orange and red color dashed lines, respectively. The spatial patterns of model-predicted and chemically-measured concentrations of Cd in wheat grain, demonstrated high consistence. The map was created by software ArcGIS 9.3.

The high consistence between the model-predicted and the analytically measured concentrations of wheat grain Cd also was extended spatially to different geographic areas of the delta region (Fig. 5). It shows that wheat grains harvested from the “Grade V” sites generally contained Cd above the safety threshold value of 1.2 μg g^−1^, whereas grains from the “Grade IV” areas contained Cd at levels in the concentration range of “Grade IV of wheat grain” per described in the national food safety standards. The high consistence further confirmed the feasibility of using the prediction models to evaluate ecological risks of soil heavy metals.

To our best knowledge, so far no scientific publications have presented results on the use of soil MIR spectral information to predict the bioaccumulation of heavy metals in plants, though the application of the MIR technique to predict Fe, Ni, Cu and Zn concentrations of soil has been studied^10^,^38^. The Fourier-transform infrared spectroscopy was successfully used to study the interactions of selenium with living bacterial cells^39^. The relatively high concentrations of Cd in the wheat grain collected from the estimated “Grade V” soil zone as described in the present study was also observed in an separate investigation by Huang *et al*.^28^. who chemically analyzed wheat grains harvested from the same area and found that most of the samples had Cd concentrations above 1.2 μg g^−1^.

### Prediction mechanism

Accumulation of heavy metals in plant body is mainly via absorption of soluble metal ions from the soil solution by plant roots, which is a selective process that requires the aid of carriers in transporting metal ions into plant tissues. In addition, the co-existence of other ionic nutrients in soil affects plant uptake of heavy metals. Therefore, an acceptable prediction model must consider the soil chemical composition and the chelation reactions between metal ions and the carrier molecules. Despite that the bands in the soil MIR spectra are not directly associated to the presence of heavy metals per se, it is clear that metals in soil interact with other existing soil components, specially the anionic functional groups through complexation reactions. These possible interactions have been explored by other researchers using IR spectroscopy^40^. For example, metal−CO exhibits an absorbance peak at 1700–2130 cm^−1^, whereas metal−CO−metal exhibits an absorbance peak at 1780–1900 cm^−1 ^19.

The DRIFTS spectra disclose useful information about metals and their chelation with chemical functional groups as influenced by the environmental conditions^8^,^9^,^13^. Previous studies reported the spectral signatures of a large array of organometallic complexes^13^. One emerging use of MIR spectra is to detect and quantify particular components in solid samples. Overall, soil DRIFTS spectra are able to reflect the effects of various soil characteristics on plant uptake of metals; the technique can be used to predict the bioavailability of heavy metals in soil. It should also be noted that the information on heavy metals complexing with anion functional groups is complicated as reflected in the MIR spectra, since mostly multiple anionic functional groups co-interact with heavy metals and mark the spectra with overlapping characteristic peaks. In this case, the PCA offers great assistance in extracting from MIR spectra the most significant factors that related to the heavy metal bioavailability.

The spectral bands adopted in the established prediction models were consistent with the bands of characteristic absorption peaks of anionic functional groups related to metal bioavailability, such as metal–SO, metal–NO, metal–CO, metal–NH, metal–C = C, and so on (Fig. S-1, SI). To be specific, the band at 486 cm^−1^ indicates the deformation vibration of Metal−Si−O or metal-carbon stretching of organometallic compounds^41^. The bands at 552 cm^−1^ and 698 cm^−1^ represent the characteristic peaks of SO_4_^2−^ and SO_3_^2−^, respectively^24^. The peak at 1042 cm^−1^ is attributed to stretching vibration of Si–O bond or C−O bond of metal carbohydrates^9^,^25^. The peaks at 814 cm^−1^, 1332 cm^−1^, and 1370 cm^−1^ demonstrate the existence of NO_3_^− ^22,24,35, and the peak at 1546 cm^−1^ represents the deformation vibration of N−H in organic acids^41^. The peaks occurring in the band window of 1640–1850 cm^−1^ are due to the presence of C = C double bonds in organic compounds^33^ or COO− in metal carboxylate^22^,^35^,^41^. The peak at 2924 cm^−1^ is of the aliphatic (C−H) bond of aliphatics^22^, whereas the peak at 3736 cm^−1^ is ascribed to O−H of mineral compounds or carbohydrates^25^. In the present study, the characteristic peaks of metal-carbonates and some silicates were rejected from the prediction models. The carbonates and silicates are two major types of soil minerals immobilizing heavy metals, and engender generally inhibiting effects on the bioavailability of heavy metals. Nevertheless, the effects remain similar for soils developed from like-parent materials in a particular region. In general, the spectral bands adopted by the established models are parallel to the characteristic absorption bands of anionic functional groups affecting the bioavailability of heavy metals, providing additional rationales of the prediction models by incorporating of soil chemical properties.

## Material and Methods

### Experimental field site

**Figure 6:**
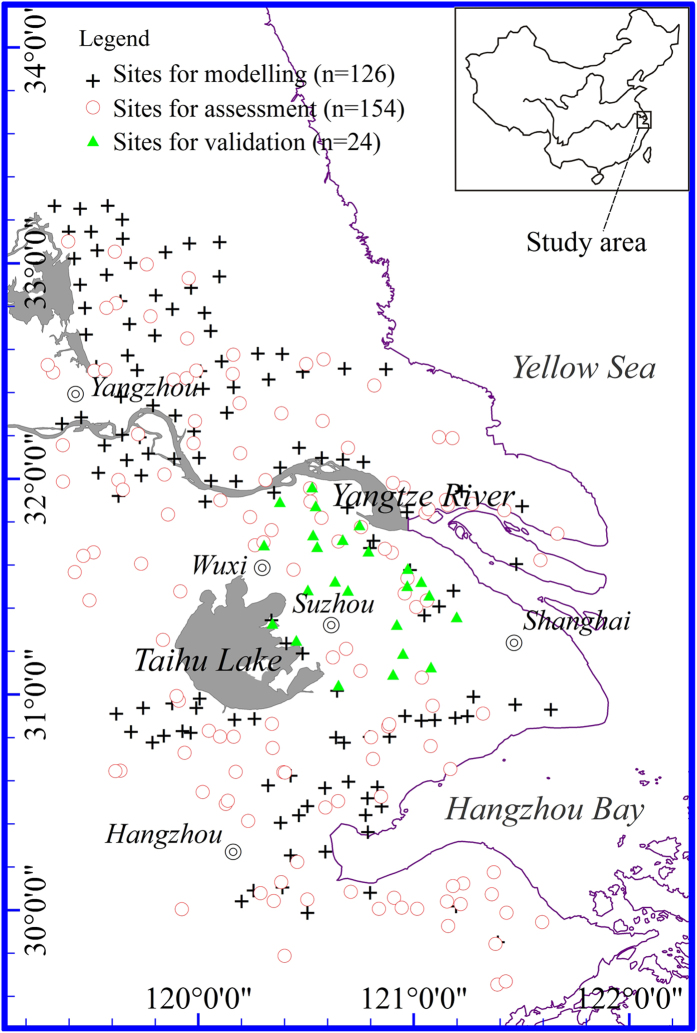


A crop rotation (wheat and rice) farming area in the Yangtze River Delta region of China was selected as the study site. The area covering 30°00′N–33°20′N and 119°10′E–121°40′E is located in the eastern China (Fig. 6). The soils in this area are mainly silt loam and clay loam developed from sedimentary rock weathering residuals and fluvial alluvial deposits. The frost-free period of the area is 210–270 days, its average annual temperature is 15 °C, and average annual precipitation is 1,000–1,400 mm. Sequential cropping of winter wheat (*Triticum aestivum*) and rice (*Oryza sativa*) on the same land in one year is a typical farming practice. Chemical fertilizers and pesticides are heavily applied to grow these two staple cereal grain crops. The irrigation water for growing rice may be contaminated by industrial waste discharges and therefore, soil contamination by heavy metals has not been rare in this area^27^,^28^,^30^.

### Soil and wheat sampling

126 pairs of soil and from which the wheat grain harvested were sampled in 2012 shortly before wheat harvesting (late May) from separate locations across the whole study site (Fig. 6). At each sampling location, soils were collected from the top 20 cm layer using a stainless steel trowel at 5 diagonal spots and mixed thoroughly to obtain a composite sample. The corresponding wheat ears were also collected from a 5 m × 5 m microplot at each of the same five spots and combined together to form a composite wheat grain sample. In 2013, additional 154 soil samples were collected randomly-selected locations in the Yangtze River Delta region. These later-collected soil samples were then characterized using DRIFTS and the spectral information was applied to on the bioavailability of heavy metals in the soils by following the pre-established, DRIFTS-based prediction models. To further assess the established soil metal bioavailability prediction models, 24 new wheat grain samples were collected in 2013 from the pre-assessed high risk zones of the delta region, chemically analyzed for heavy metal concentrations, and the results were compared with the values estimated through the prediction models.

In the laboratory, the soil samples were air-dried and ground to pass a 2-mm sieve. Aliquots of the <2 mm samples were further ground using an agate mortar and separated by a nylon sifter, the particles of <74 μm was stored for chemical characterization. The wheat ears were manually threshed and the grains were rinsed with deionized water, air-dried, and ground to <74 μm. Prior to chemical analysis, the wheat grain powders were oven-dried at 60 °C for 48 hours to remove any moisture.

### Chemical analyses

The pH values of the soils were measured in 1:2.5 unground soil /water (w/v) slurries using a pH meter equipped with a combination electrode (Model PHS-3C, Shanghai Precision and Scientific Instrument Co. Ltd., China). The slurries were prepared by adding 25 mL of deionized water to 10 g of <2 mm air-dry soil in a 50-mL beaker and manually agitating the mixture for 5 minutes. The procedures of soil pH measurement followed the protocol described by Thomas^42^, with adjustment of the suggested soil /water (w/v) ratio from 1:1 to 1:2.5. The total concentrations of Ca, Mg, K, Fe, S and P in the soil samples were determined by the powder X-ray fluorescence (XRF; ZSX primus II, Rigaku Japan) technique. Soil organic carbon (OC) content was determined following the modified Tyurin dichromate oxidation methods^43^. The soil total N content was measured following the Kjeldahl method with H_2_SO_4_ digestion^44^. To measure heavy metal contents, aliquots of the <74 μm soil samples were digested with HCl-HNO_3_-HClO_4_-HF^45^. The acid digestates were then analyzed for Ni, Cu, Cr, Zn and Mn concentrations using an inductively coupled plasma-optical emission spectrometer (ICP-OES; Thermo Element iCAP6000 (Radial), Cambridge, BZ, UK) and for Cd and Pb concentrations using an inductively coupled plasma mass spectrometer (ICP-MS; Thermo Element X Series 2, Bremen, Germany). To determine the Hg concentration, soil samples were first digested with aqua-regia^46^ and then analyzed using the hydride generation atomic fluorescence spectrometry (HG-AFS; AFS-3100, China).

The wheat grain powder samples were digested with HNO_3_ and H_2_O_2_^47^, and then analyzed for concentrations of Cd, Pb, Ni, Cr, Cu, Zn and Fe using ICP-MS and that of Hg using the HG-AFS method.

Quality assurance and quality control were implemented by including procedure blanks, duplicate samples, and certified reference materials (approved by the General Administration of Quality Supervision, Inspection and Quarantine of the People’s Republic of China) in the chemical analyses. Elemental recoveries and relative standard deviations (RSDs) of the certified reference materials analysis were 96–105% and <5.0%, respectively.

### Spectral analyses

The <74 μm soil samples were placed in a specialized macro sample cell and their DRIFTS spectra were collected using a Nicolet 6700 IRFT spectrometer (Thermo Scientific, Waltham, MA, USA) equipped with a Spectra-Tech Collector II diffuse accessory. The spectral resolution value was set at 2.0 cm^−1^. The reflectance intensity of each sample relative to a KBr sample was recorded directly on a computer using OMNIC software (Version 8.2, Thermo Electron Corporation, Waltham, MA, USA). The reflectance (ref) data were transformed into Kubelka-Munk units [K-M = (1 − ref)^2^/(2 × ref)]. To minimize additive baseline effects, the first and second derivative spectra of the transformed reflectance data were calculated using Savitzky-Golay smoothing with a second-order polynomial fit on these spectral responses (reflectance and K-M transformed), a function of the Unscrambler software (version 8.0.5, CAMOASA, Trondheim, Norway).

### Data analyses and modeling

Since not all spectral bands were adequately sensitive to reflect the bioavailability of heavy metals in soils or the concentrations of heavy metals in wheat grain. Thus, the Spearman correlation analysis was conducted first to find out the bands of soil DRIFTS spectra highly sensitive to the bioavailable portion of soil heavy metals that could be accumulated in wheat grain. This aim was accomplished by evaluating the Spearman correlation coefficients for linear regression analysis of the soil DRIFTS reflectance against the corresponding wheat grain heavy metal concentrations. As the yielded sensitive bands usually covered a broad range of wavenumber, the most representative spectral bands were extracted subsequently via principal component analysis (PCA). The extracted principal components (PCs) were the factors possessing the closest relations with the targeted variable (here is heavy metal in wheat grain)^48^,^49^. Thus, PCs are usually regarded as potential predictors of the target variable, illustrating the physical knowledge about the inter-relation between factors and target variable^8^. The PCA also gave the standardized factor score of each PC. To establish prediction models for heavy metals in wheat grain based on soil DRIFTS reflectance, the concentration data of heavy metals in wheat grain was regressed against the standardized factor scores of the PCs by the multiplelinear regression analysis^50^.

To optimize and simplify the prediction model, only the representative peak bands at which the reflectance of soil DRIFTS spectra demonstrated the highest correlation coefficient with heavy metal concentration (*p* < 0.01) was chosen from each PC-corresponding spectral band region. The established models were applied to predict concentrations of heavy metals in wheat grains from the mid Yangtze River Delta region based on the DRIFTS spectra of the soils from the same area. The prediction results were compared with the chemically measured values to validate the models.

Spectral data processing and multivariate statistical analysis were performed using OMNIC 8.2 and SPSS packages (Version 18, SPSS Inc., Armonk, NY, USA), respectively.

### Geostatistics

The planar distribution of the predicted concentrations of Cd in wheat grain was performed by ordinary kriging spatial interpolation. First, the established prediction models were exercised to estimate the concentration of Cd in wheat grain based on the DRIFTS spectral results of soil samples collected from 154 sites. Second, based on the 154-sites data, Cd concentration of wheat grain at the un-sampled sites was calculated with the geostatistical analysis. Information generated through semivariogram was used to calculate sample-weighing factors for spatial interpolation by an ordinary kriging procedure in the Geostatistical Analysis extension in ArcGIS (Version 9.3), and the detailed information can be found in the Supplementary Information.

## Additional Information

**How to cite this article**: Wang, C. *et al*. Ecological risk assessment on heavy metals in soils: Use of soil diffuse reflectance mid-infrared Fourier-transform spectroscopy. *Sci. Rep.* **7**, 40709; doi: 10.1038/srep40709 (2017).

**Publisher's note:** Springer Nature remains neutral with regard to jurisdictional claims in published maps and institutional affiliations.

## Supplementary Material

Supplementary Information

## Figures and Tables

**Table 1:** Prediction models for wheat grain heavy metal concentrations based on the adopted spectral bands (characteristics peaks) of soil DRIFTS.

Model	R^2^	RMSE
Fe = 163.44 − 1.854Ref_486_ − 1.836Ref_1424_ − 2.154Ref_1546_ + 20.42Ref_1632_ − 30.056Ref_1642_ + 18.809Ref_1658_ − 6.517Ref_1736_ + 3.157Ref_1832_ − 0.907 Ref_2360_	0.378	11.9
Cu = 17.393 − 0.118Ref_486_ − 0.197Ref_1424_ + 0.171Ref_1546_ + 1.165Ref_1632_ − 1.169Ref_1642_ + 0.346Ref_1658_ − 0.361Ref_1736_ + 0.085Ref_1832_ + 0.049Ref_1924_ − 0.126 Ref_2360_	0.374	1.05
Zn = 93.953 − 0.491Ref_486_ − 2.413Ref_1424_ + 0.461Ref_1546_ + 9.409Ref_1632_ − 11.691Ref_1642_ + 6.307Ref_1658_ − 5.025Ref_1736_ + 5.486Ref_1832_ − 2.271Ref_1924_ − 0.551 Ref_2360_	0.364	7.93
Hg = 4.798 − 0.073Ref_486_ + 0.165Ref_1424_ − 0.178Ref_1546_ − 0.127Ref_1632_ − 0.1Ref_1642_ + 0.504Ref_1658_ − 0.082Ref_1736_ − 0.496Ref_1832_ + 0.462Ref_1924_ − 0.108 Ref_2360_ + 0.056Ref_3736_	0.399	0.39
Cd = 0.218 − 0.001Ref_552_ − 0.031Ref_698_ + 0.033Ref_814_ + 0.005Ref_1042_ − 0.019Ref_1370_ + 0.004Ref_1546_ + 0.01Ref_1722_ − 0.001Ref_1868_ − 0.0004 Ref_2360_ − 0.006Ref_2924_	0.327	0.02
Ni = 0.724 − 0.001Ref_552_ + 0.161Ref_698_ − 0.11Ref_814_ − 0.055Ref_1042_ − 0.035Ref_1332_ + 0.043Ref_1546_ − 0.038Ref_1722_ + 0.022Ref_1868_	0.312	0.08

Ref_i_, reflectance at band of i. RMSE, the root mean square error. The units for metal concentrations are μg g ^−^ ^1^, except for Hg is μg kg ^−^ ^1^.
