# Supplementary material for: Ecological risk assessment on heavy metals in soils: Use of soil diffuse reflectance mid-infrared Fourier-transform spectroscopy
Source: Sci Rep. 2017 Feb 13;7:40709. doi: 10.1038/srep40709 (PMC5304163; doi:10.1038/srep40709)
Supplement: Supplementary Information [file srep40709-s1.pdf]

Supplementary Information

Ecological risk assessment on heavy metals in soils: Use of soil diffuse reflectance mid-infrared Fourier-transform spectroscopy

Cheng Wang,[1,2] Wei Li,[2] Mingxing Guo,[3] Junfeng Ji[*,2]

[1] Jiangsu Key Laboratory of Atmospheric Environment Monitoring and Pollution Control, Collaborative Innovation Center of Atmospheric Environment and Equipment Technology, School of Environmental Science and Engineering, Nanjing University of Information Science & Technology, Nanjing, 210044, China

[2] Key Laboratory of Surficial Geochemistry, Ministry of Education, School of Earth Sciences and Engineering, Nanjing University, Nanjing 210093, China

[3] Department of Agriculture & Natural Resources, Delaware State University, Dover, DE 19901, USA

This supporting information consists of 9 pages, including three tables, two figures and literature cited.

The detail of risk assessment on Cd in arable soil using soil DRIFTS-based prediction model

Safety grade criterion

For food safety, wheat grain Cd concentration is divided into five grades (Table S-3) based on the Chinese Maximum Permissible Concentrations (MPC) for heavy metals in wheat grain.[1] Accordingly, the soil was also divided into five corresponding grades for wheat grain production (Table S-3). For example, a Grade I soil means that the produced wheat grain at this site should present Cd concentration lying in the range of Grade I of wheat grain (values in Row No. 3, Table S-3)

Prediction of spatial distribution of wheat grain Cd concentration and mapping the result of ecological risk assessment

154 soil samples distributed in the Yangtze River delta region were collected to gain the diffuse reflectance mid-infrared Fourier-transform spectra (DRIFT). Then the Cd concentration of suppositional corresponding wheat grain grown at these sites was estimated using the established Prediction Model in Table 1 (in the text).

Based on the predicted wheat grain Cd concentrations of 154 sites, estimation and mapping of Cd concentration of wheat grain at the un-sampled sites was calculated with the geostatistical analysis according to Liu et al.[2] Geostatistics provides a set of statistical tools for incorporating spatial coordinates of data processing, which had proved useful in mapping spatial variability at un−sampled sites.[3] Kolmogrov–Smirnov (K–S) test for goodness-of-fit was firstly performed to test whether the

dataset for predicted Cd value was normally distributed,[4] and the result is positive. Semivariograms were constructed using GS+ (Version 9.0) to examine the degree of spatial continuity of Cd among data points and to establish their range of spatial dependency. The results show that predicted Cd had a greater variation, with a CV of 107%. The wheat grain Cd was best fit with a spherical model. Information generated through semivariogram was used to calculate sample-weighing factors for spatial interpolation by an ordinary kriging procedure [5-7] in the Geostatistical Analysis extension in ArcGIS (Version 9.3).

Based on the spatial distribution map of the predicted Cd concentration that generated from the kriging interpolation, the risk assessment result was plotted (Fig. 4 in the text) by introducing the assessment criterion in Table S-3 into the data classification.

References

1. Ministry of Health of China. *Maximum Level of Contaminants in Food (GB2762-2012).* (Chinese National Standard Agency, Beijing, 2012).
2. Liu, X., Wu, J. & Xu, J. Characterizing the risk assessment of heavy metals and sampling uncertainty analysis in paddy field by geostatistics and GIS. *Environ. Pollut.***141** (2), 257–264 (2006).
3. Goovaerts, P. Geostatistics in soil science: state-of-the-art and perspectives. *Geoderma*, **89**(1), 1–45 (1999).
4. Sokal, R. R. & Rohlf, F. J. *Biometry: the principles and practice of statistics in biological research, 2nd.*, 83–123(Freeman, San Francisco, 1981).
5. Isaaks, E. H. & Srivastava, R. M. *An introduction to applied geostatistics.* (Oxford University Press, New York, 1989).
6. Lee, C. S., Li, X., Shi, W., Cheung, S. C. & Thornton, I. Metal contamination in urban, suburban, and country park soils of Hong Kong: a study based on GIS and multivariate statistics. *Sci. Total Environ.* **356**(2), 45–61 (2006).
7. Yang, Y. & Christakos, G. Uncertainty assessment of heavy metal soil contamination mapping using spatiotemporal sequential indicator simulation with multi-temporal sampling points. *Environ. Mon. Ass.*187, 571 (2015).

Table S-1 Concentrations of heavy metals in soil and wheat grain and some soil parameters

	Soil		Wheat grain	
	Mean	Range	Mean	Range
Cd ($\mu g\ g^{-1}$)	0.215	0.080-1.249	0.065	0.018-0.245
Cr ($\mu g\ g^{-1}$)	75.2	36.4-93.5	0.11	0.04-0.37
Cu ($\mu g\ g^{-1}$)	31.5	14.9-70.9	7.13	4.02-17.75
Hg ($\mu g\ g^{-1}$)	0.171	0.030-0.741	1.89[a]	0.40-6.6[a]
Ni ($\mu g\ g^{-1}$)	30.1	10.2-55.2	0.29	0.07-1.11
Pb ($\mu g\ g^{-1}$)	18.4	6.9-54.0	0.080	0.022-0.109
Zn ($\mu g\ g^{-1}$)	88.8	34.1-229.6	41.8	20.9-108.6
Fe ($g\ kg^{-1}$)	32.86	10.07-45.01	70.3	34.0–193.0
pH	6.72	4.80-8.28	/	/
OC ($g\ kg^{-1}$)	20.17	4.60-36.59	/	/
N ($g\ kg^{-1}$)	1.78	0.51-3.53	/	/
P ($g\ kg^{-1}$)	0.93	0.43-2.17	/	/
K ($g\ kg^{-1}$)	18.08	0.64-24.32	/	/
Ca ($g\ kg^{-1}$)	12.41	1.57-40.17	/	/
Mg ($g\ kg^{-1}$)	9.46	1.81-14.29	/	/
Mn ($g\ kg^{-1}$)	0.57	0.15-1.05	/	/
S ($g\ kg^{-1}$)	0.39	0.17-0.76	/	/

[a], the unit is $\mu g\ kg^{-1}$.

Table S-2 Prediction models of wheat grain heavy metals concentrations based on the factor score of principal component using the principal component regression analysis

	Model	R^2	RMSE(%)
Fe	=70.277−10.279FS1−5.021FS2+0.828FS3+0.26FS4−1.339FS5+1.102FS6 −4.908FS7+3.82FS8−3.114FS9−0.933FS10	0.314	12.3
Cu	=7.135−0.786FS1−0.648FS2−0.026FS3+0.032FS4+0.026FS5−0.064FS6 −0.601FS7+0.104FS8−0.365FS9−0.081FS10	0.375	1.07
Zn	=41.789−4.677FS1−2.401FS2−0.556FS3−1.224FS4−2.655FS5+1.456FS6 −3.285FS7+1.346FS8−1.353FS9−0.591FS10	0.315	7.95
Hg	=1.894−0.390FS1−0.348FS2−0.054FS3+0.197FS4−0.337FS5+0.044FS6 +0.067FS7−0.11FS8−0.417FS9−0.006FS10−0.152FS11	0.406	0.39
Cd	=0.065−0.008FS1−0.008FS2−0.003FS3−0.003FS4−0.008FS5+0.007FS6 −0.005FS7−0.001FS8+0.008FS9−0.0003FS10+0.01FS11	0.351	0.02
Ni	=0.292−0.055FS1−0.037FS2+0.008FS3−0.081FS4−0.005FS5+0.051FS6 +0.02FS7+0.005FS8	0.367	0.09

FSi, the standardized factor score of principal component i. RMSE, the root mean square error. The units of metals are μg g^{-1}, except of Hg is μg kg^{-1}.

Table S-3 Safety grade criterion of the wheat planting system (for Cd)

Wheat grain				Arable soil	
Grade	According as	Range of Cd concentration of wheat grain (μg g^{-1})		Grade[b]	Corresponding soil risk assessment
I	Concentration[a] below 60% of MPC	<0.06		I	Safe for wheat production
II	Concentration[a] lower than the MPC, but higher than 60% of MPC	0.06-0.08		II	Safe for planting wheat
III	Concentration[a] lower than the MPC, but higher than 80% of MPC	0.08-0.10	⟺	III	Still can plant wheat, but should strengthen monitoring
IV	Concentration[a] higher than the MPC, but the excess <20%	0.10-0.12		IV	Not suitable for planting wheat
V	Concentration[a] exceeds the MPC, and the excess ≥20%	≥0.12		V	Obviously unsafe for wheat production

[a], the concentration herein is the Cd concentration of wheat grain. MPC, the Chinese Maximum Permissible Concentrations of Cd in wheat grain. [b], the corresponding estimated grades of soil site, is matched with the classification of wheat grain Cd concentration, i.e., the “Grade I soil” means that the produced wheat grain at this grade soil will present Cd concentration lie in the range of “Grade I of wheat grain” (values in Row No. 3).

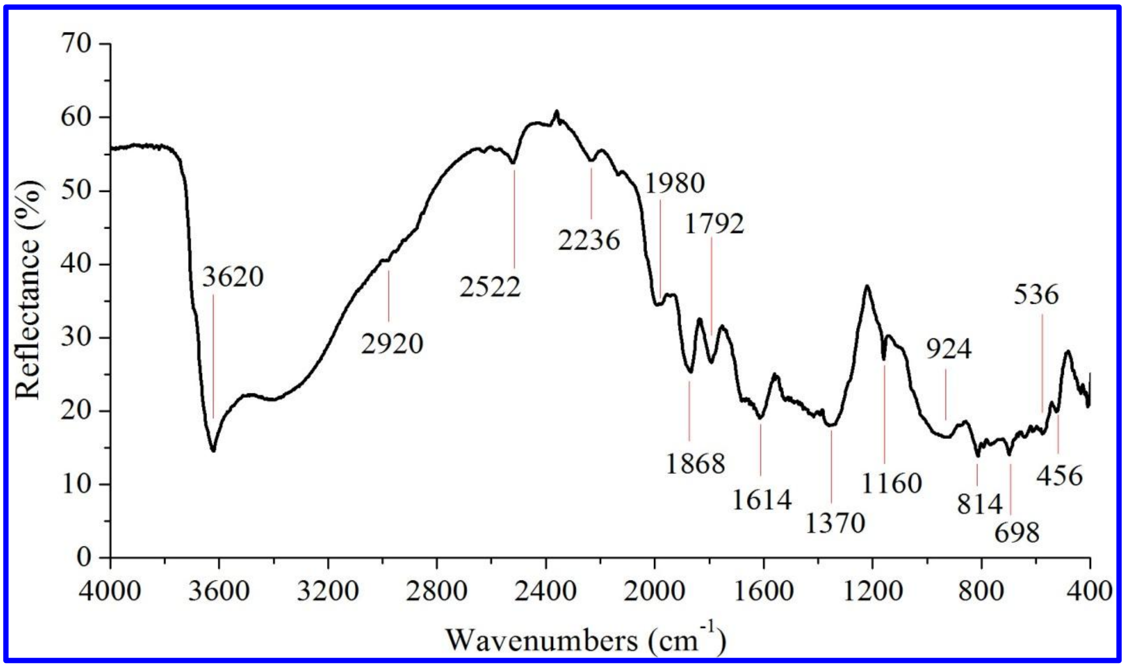

Figure S-1 Typical diffuse reflectance mid-infrared Fourier-transform spectrum of arable soil in the Yangtze River Delta area

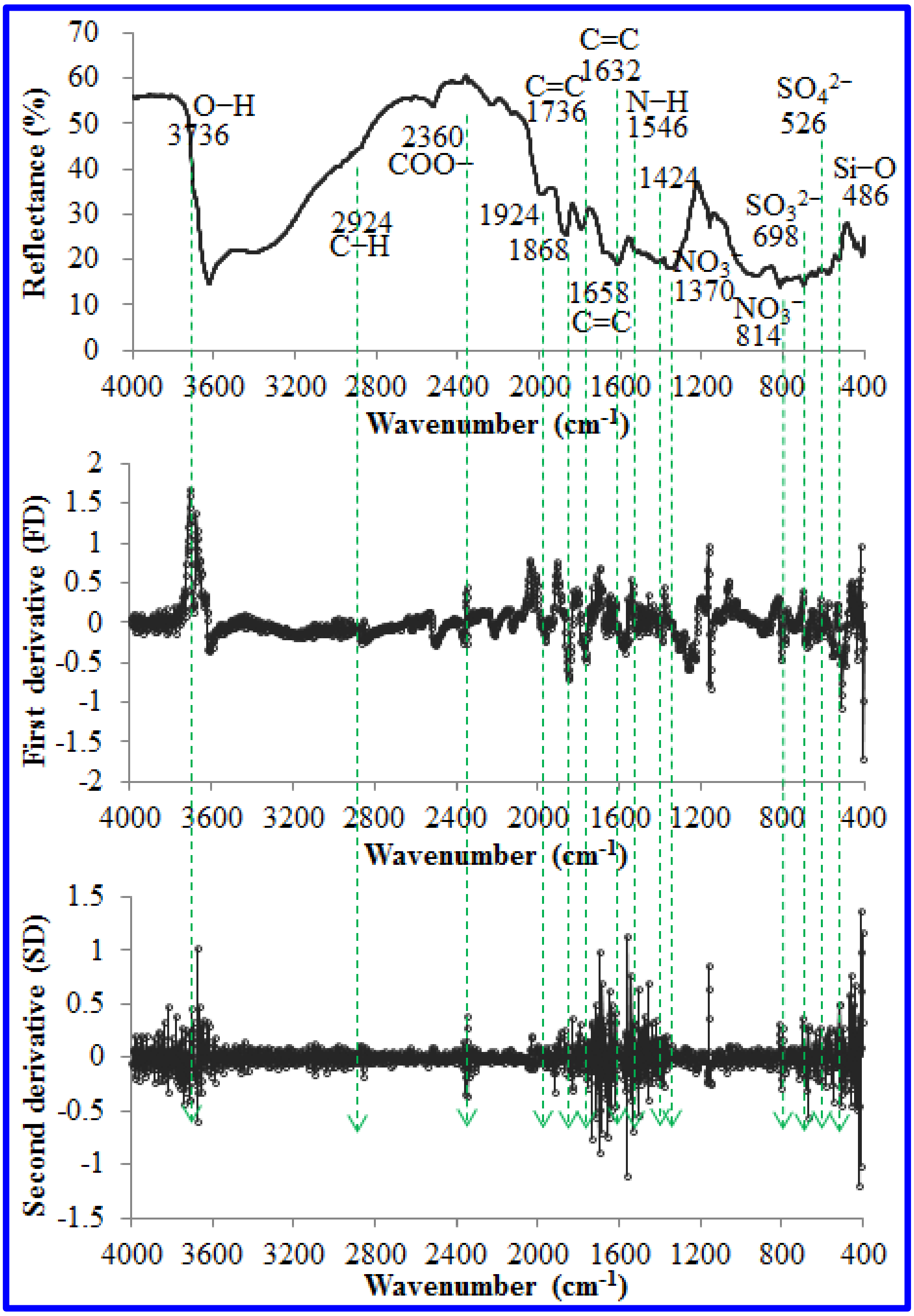

Figure S-2 Adopted band in prediction model and their corresponding absorption peaks. The adopted spectra bands in the present prediction model are consistent with the characteristic peaks of coupled anions or molecular functional groups, respectively.
